# Bilateral Infiltrating Ductal Carcinoma With Adrenal Metastasis: A Rare Case Report

**DOI:** 10.7759/cureus.65635

**Published:** 2024-07-29

**Authors:** Pragna Puvvada, Dakshayani S Nirhale, Romi H Gaudani, Praveen Mane

**Affiliations:** 1 General Surgery, Dr. D. Y. Patil Medical College, Hospital & Research Centre, Dr. D. Y. Patil Vidyapeeth (Deemed to be University) Pimpri, Pune, IND

**Keywords:** adrenal glands, chemotherapy, adrenalectomy, metastasis, invasive ductal carcinoma

## Abstract

Adrenal gland metastasis is rare, often associated with invasive lobular carcinoma (ILC) rather than infiltrating ductal carcinoma (IDC). This report presents a case of a 43-year-old female with bilateral breast IDC and metastasis to the adrenal gland, with bilateral axillary and supraclavicular node involvement. Initial symptoms included a nipple discharge and a palpable lump. Diagnostic imaging and biopsy confirmed IDC, grade 2, with positive estrogen receptor (ER) and progesterone receptor (PR) status and negative human epidermal growth factor receptor 2 (HER2) status. The patient is undergoing chemotherapy and radiotherapy, with adrenalectomy planned post chemotherapy. The case underscores the need for early diagnosis and rapid treatment to improve outcomes, highlighting the paucity of data on managing solitary adrenal metastasis from IDC. Further research and clinical trials are essential to develop standardized treatment protocols.

## Introduction

The most prevalent cancer in females to be diagnosed is breast cancer, which also ranks second globally in terms of cancer-related deaths in females [1]. The most common type of breast cancer is infiltrating ductal carcinoma (IDC), which accounts for 70%-85% of all invasive breast cancer cases [2]. It arises from the cells lining the milk ducts and invades surrounding tissues, making it a potentially aggressive and life-threatening disease [3]. IDC typically presents as a painless lump or thickening in the breast, although other symptoms such as changes in skin texture or nipple discharge may also occur [4]. Although metastasis to the adrenal gland is exceedingly uncommon, breast cancer typically spreads to the lung, liver, bone, and brain. Breast cancer metastases frequently result in a worse prognosis and a lower five-year survival rate [5]. Breast cancer adrenal metastases are typically linked to invasive lobular carcinoma (ILC), which most frequently occurs concurrently with multiorgan metastases. Breast carcinomas with isolated adrenal metastases are uncommon, particularly when the carcinomas start from intraductal carcinomas [6].

The tumor size, grade, hormone receptor status, and existence of lymph node involvement are among the variables that affect a patient's prognosis when they have invasive ductal carcinoma [4]. The best course of action for a single adrenal metastasis from breast cancer is still unknown due to its rarity. Palliative chemotherapy is typically advised when a cancer patient has distant visceral metastases, as this can be distressing [2]. Improving outcomes for people with this disease requires early diagnosis through screening mammography and rapid treatment.

We present a rare case of the metastasis of IDC to the adrenal gland with bilateral breast and axillary node involvement with supraclavicular activity managed by chemotherapy and radiotherapy and planned for adrenalectomy after evaluating the patient post chemotherapy.

## Case presentation

**Figure 1:**
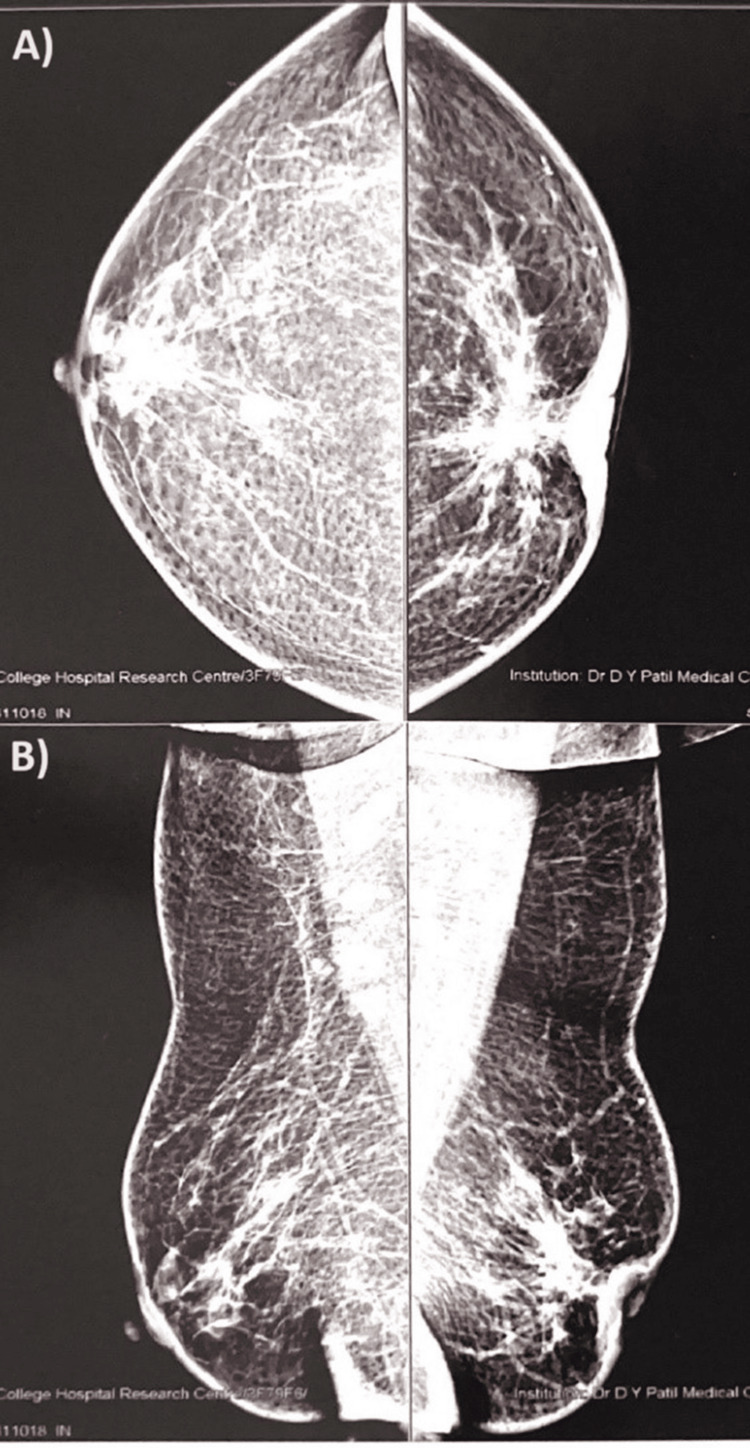
Mammography of the bilateral breast with axillary panel A) Bilateral breast showing irregular dense masses. B) Bilateral breast with the axillary panel showing bilateral axillary lymphadenopathy

A 43-year-old female presented to our surgery department with complaints of discharge from her left nipple for a month and a lump in her left breast for three months, with type 2 diabetes mellitus for three years. On physical examination, circumferential nipple retraction was observed in the left breast, and a 3×3 cm hard lump was palpable posterior to the nipple-areolar complex, hard mobile bilateral axillary lymph nodes were palpable, and a left supraclavicular node was palpable, which prompted further investigation. No similar family history was noted. On the full-field digital mammography with adjunct USG, a large hypoechoic irregular mass was found in the central mammary region with significant internal vascularity extending and involving the nipple-areolar complex in the left breast, whereas a large hypoechoic irregular mass with angulated margin and significant internal vascularity was found in the right breast; the mammography revealed an irregularly defined dense mass measuring 68×33 mm in the retroareolar and central mammary region in the left breast and an irregular mass lesion measuring 8×9 mm in the central area of the right breast and bilateral axillary lymphadenopathy with bulky thickened cortex, which are likely metastatic nodes (Figure 1).

**Figure 2:**
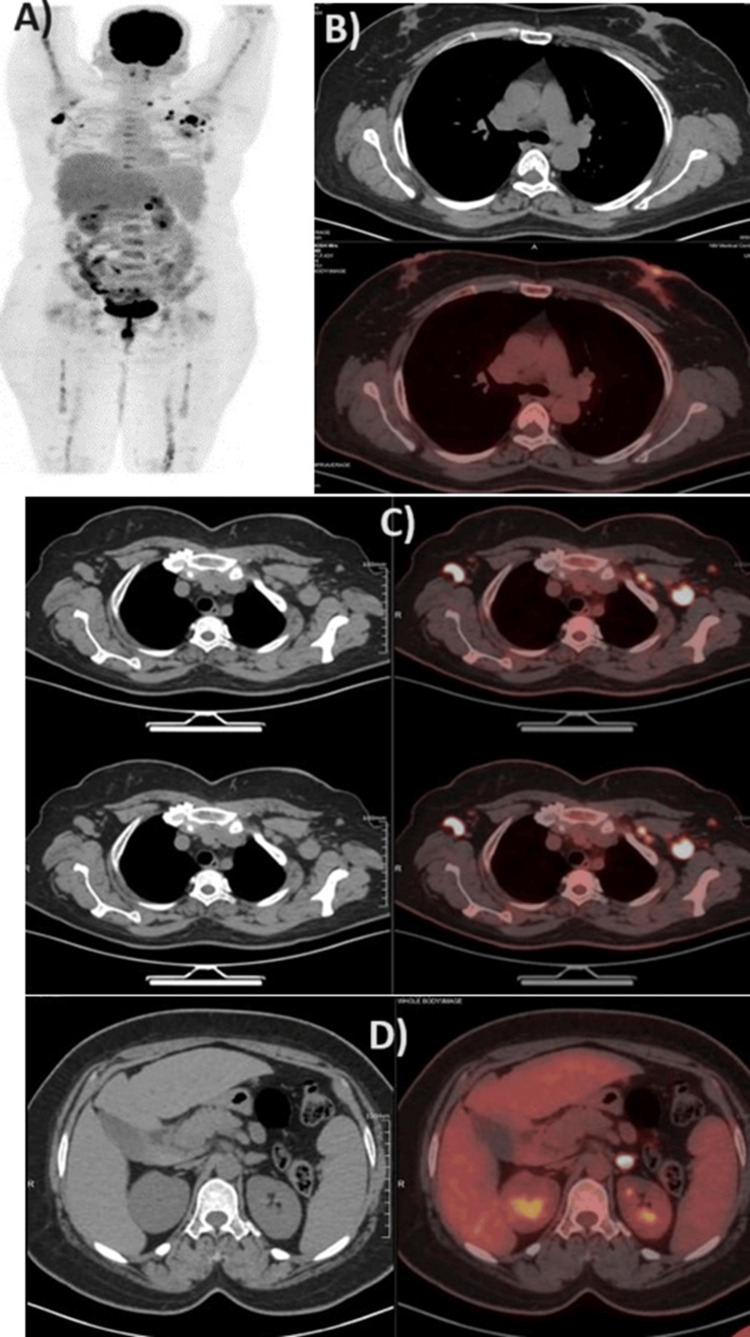
Radiology images confirming the diagnosis A) PET scan of the whole body. B) Section with reactive bilateral breast with nipple retraction. C) Section with reactive lymph nodes. D) Section with high intensity in the left adrenal gland

The PET scan revealed a mild metabolically active 1.6×1.4 cm retroareolar lesion in the left breast parenchyma with the involvement of the nipple-areolar complex representing primary malignancy (maximum standardized uptake value {SUVmax}: 4) along with a discrete 8 mm rounded nodular lesion in the upper quadrant of the contralateral right breast (SUVmax: 7.9) representing synchronous primary malignancy. Enlarged multiple bilateral axillary lymph nodes are identified with the largest being 2.5×2.4 cm (SUVmax: 11.5) in the left axilla and 2.5×1.3 cm (SUVmax: 12) in the right axilla, which indicate metastatic status. A mildly enlarged metastatic metabolically active 1.1 cm level 4 left cervical lymph node with SUVmax of 7.5 is identified. Also, a discrete 1.1×0.8 cm rounded nodular lesion is identified in the left adrenal gland with focal increased metabolic activity, most likely to be metastatic (SUVmax: 14.2) (Figure 2).

**Figure 3:**
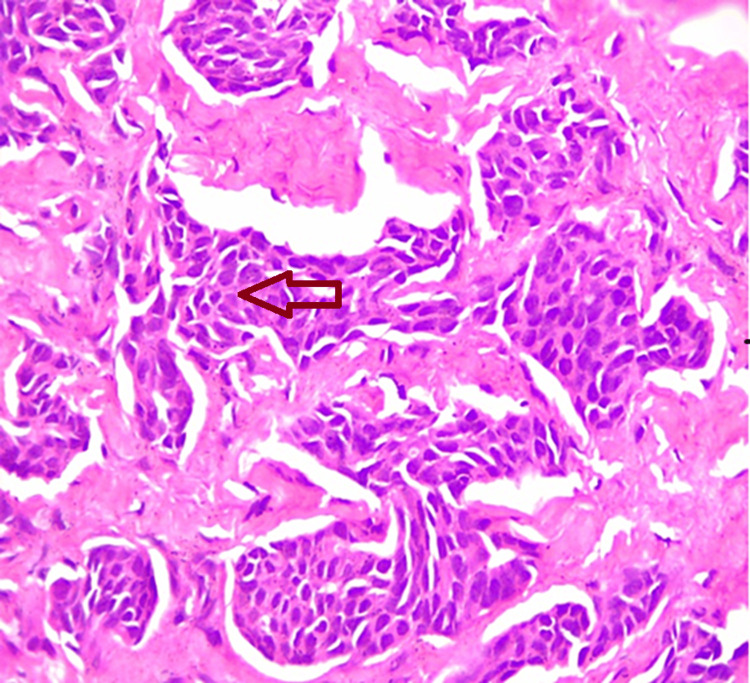
Histopathological imaging showing invasive ductal carcinoma Hematoxylin and eosin staining showing the invasive ductal carcinoma cells (arrow), using 100× high-power magnification

Further histopathology imaging studies revealed a suspicious mass, and a subsequent biopsy confirmed the diagnosis of infiltrating ductal carcinoma grade 2 (Figure 3).

**Figure 4:**
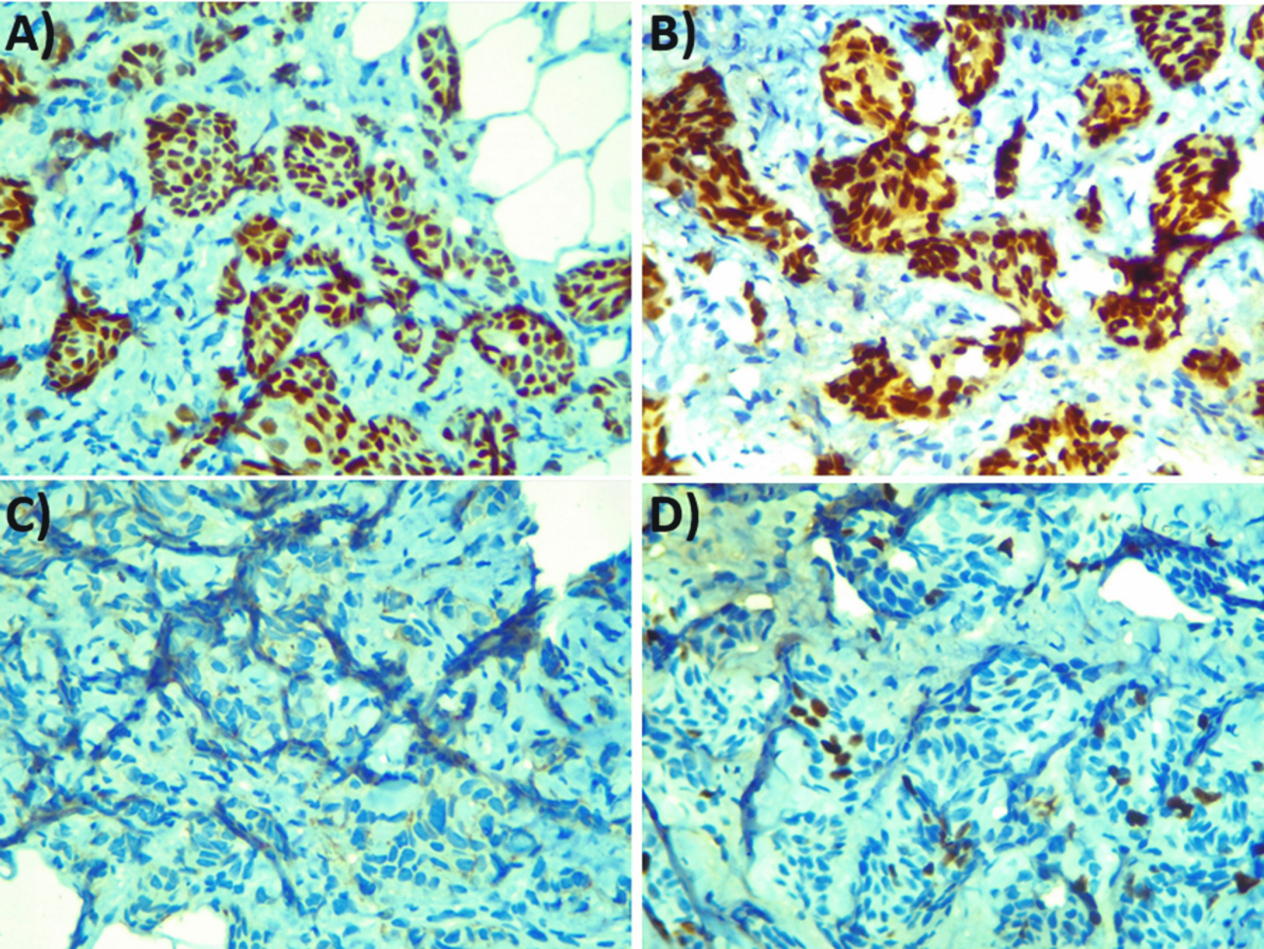
Immunohistochemical staining images A) Novocastra straining used, showing PR positivity (100×, high power). B) PR636 staining used, showing ER positivity (100×, high resolution). C) HercepTest kit (Agilent Technologies, Santa Clara, CA) staining used, showing HER2 negativity (100×, high resolution). D) MIB-1 staining used, showing Ki67 positivity (100×, high resolution) PR, progesterone receptor; ER, estrogen receptor; HER2, human epidermal growth factor receptor 2

The immunohistochemistry (IHC) markers show positive for estrogen receptor (ER) and progesterone receptor (PR), have a Ki67 proliferation index of 8%-10%, and are negative for human epidermal growth factor receptor 2 (HER2) (Figure 4)

Due to the metastasis of IDC to the adrenal gland with bilateral breast and axillary node involvement with supraclavicular activity, the patient is receiving chemotherapy and is followed up every month for additional care.

## Discussion

The characteristic feature of the infiltrating ductal carcinoma (IDC) of the breast is the invasion of surrounding breast tissue, which may potentially spread to different body parts. There exist multiple subtypes of breast cancer according to IDC classification: tubular carcinoma, medullary carcinoma, mucinous carcinoma, papillary carcinoma, and cribriform carcinoma. The risk factors associated with IDC include age, family history, hormonal factors, and lifestyle choices [7]. The metastasis of the adrenal glands is a common autopsy finding and is most common in patients with renal, lung, and gastrointestinal carcinomas. It is not common for the IDC of the breast to cause adrenal metastases. Even less common is a solitary, spontaneous, metachronous adrenal metastasis from the IDC [2]. Our patient may be the first with a long-term survival description to present with axillary and supraclavicular involvement with solitary adrenal metastases from the IDC of the bilateral breast. As of right now, there are no recommendations for the management of patients with solitary adrenal metastases of the IDC.

The prognosis for mixed breast mucinous carcinoma with a five-year survival rate of 76% is poor, and it can develop distant metastases if there is nodal involvement [5]. In general, primary cancer and adrenal gland metastases can be detected early on with a magnetic resonance imaging (MRI) or CT scan. Furthermore, adrenal metastases can be successfully detected by a PET scan. However, the specificity of these image-based detection methods has always been a problem, although these imaging techniques distinguish metastases from an original adrenal tumor. Metastasectomy or fine-needle aspiration biopsy should be used to determine the final diagnosis. Breast-specific antigens, such as mammaglobin and gross cystic disease fluid protein 15 (GCDFP-15), are now widely utilized to aid in the diagnosis of metastatic tumors from breast carcinoma. These antigens are recognized markers for epithelia of breast origin [2].

Enhancing the quality of life and extending survival are the main objectives of treating metastatic breast cancer patients. Because of its benefits, which include a quicker recovery time and an earlier hospital discharge, laparoscopic adrenalectomy (LA) has emerged as the preferred treatment for adrenal gland tumors. Patients with isolated adrenal metastases may undergo LA treatment; however, the size of the tumor limits this course of action. The following circumstances typically require surgical excision: There is only one metastatic lesion that can be excised or well controlled, and there are no metastases in other organs, and the patient is generally well and able to withstand surgery [5]. Additionally, research has focused on identifying biomarkers and genetic mutations that may play a role in the development and progression of IDC. Treatment options for IDC have also been extensively studied, with a combination of surgery, chemotherapy, radiation therapy, and targeted therapies being commonly used. In comparison to lumpectomy, a study found that adding radiation therapy reduced the incidence of ipsilateral breast cancer by 50% [8]. Adjuvant therapy for early-stage or advanced breast cancer has shown a 25% increase in overall survival rates and a 23% improvement in disease-free survival. Adjuvant therapy recommendations are based on the risk profile of each patient and the trade-off between absolute benefit and toxicity. The best treatment plans involve anthracycline-based regimens, and adding taxanes improves the prognosis for individuals with lymph node-positive illness [2]. In a study comparing the survival rates of invasive lobular carcinoma (ILC) and IDC, 87% of the patients underwent mastectomy and axillary nodal evaluation, whereas 73% received postoperative radiotherapy in the locoregional area, while 8.7% of the patients received adjuvant therapy, and 4.5% of them had ovarian ablation [9]. For individuals with hormone-receptor-positive advanced breast cancer, endocrine therapy is also a preferable alternative [2]. Recently, an extensive amount of research has been put into processing many components of automated breast cancer diagnosis, such as images from breast cytology and histology, as well as images from magnetic resonance imaging (MRI) and mammography [10].

In this particular case, adrenalectomy or surgical excision was not preferred due to the metastasis of IDC to the adrenal gland with bilateral breast and axillary node involvement with supraclavicular activity; the patient was started on chemotherapy and radiotherapy.

## Conclusions

This is one of those rare cases with an asymptomatic adrenal metastasis discovered in the metastatic workup of a diagnosed case of bilateral breast infiltrating ductal carcinoma, with bilateral axillary and supraclavicular nodal involvement. The management of adrenal metastasis in a case of bilateral breast infiltrating ductal carcinoma is still a topic of dilemma among oncology specialists, with the limited available data on the topic; a combination of adrenalectomy and chemotherapy or adrenalectomy alone has been employed on the patients, which showed promising prognosis. We propose that further investigations, incorporating clinical trials, will yield an effective approach.
